# Risk factors for breakthrough vitreous hemorrhage after intravitreal tissue plasminogen activator and gas injection for submacular hemorrhage associated with age related macular degeneration

**DOI:** 10.1371/journal.pone.0243201

**Published:** 2020-12-03

**Authors:** Jun Hyun Lim, Yong Seop Han, Sang Joon Lee, Ki Yup Nam

**Affiliations:** 1 Department of Ophthalmology, Kosin University Hospital, Busan, South Korea; 2 Department of Ophthalmology, College of Medicine, Gyeonsang National University, Jinju, South Korea; 3 Department of Ophthalmology, Gyeonsang National University Changwon Hospital, Changwon, South Korea; 4 Department of Ophthalmology, College of Medicine, Kosin University, Busan, South Korea; 5 Department of Ophthalmology, College of Medicine, Chungnnam National University, Daejeon, South Korea; Universite de Rouen, FRANCE

## Abstract

**Purpose:**

We investigated risk factors for breakthrough vitreous hemorrhage (VH) after an intravitreal tissue plasminogen activator (tPA) and gas injection in patients with submacular hemorrhage (SMH) associated with age-related macular degeneration (AMD).

**Methods:**

The medical records of patients diagnosed with SMH associated with AMD who received an intravitreal tPA (50 μg/0.05 mL) and perfluoropropane gas (0.3 mL) injection were reviewed retrospectively. We analyzed the associations of breakthrough VH with age, sex, best-corrected visual acuity, intraocular pressure, AMD subtype, accompanying sub-retinal pigment epithelium (RPE) hemorrhage, history of cataract surgery, history of hypertension and diabetes mellitus, history of drinking and smoking, and history of antiplatelet or anticoagulant medication. We also examined the relationships between various parameters, including the area ratio of the SMH to the optic disc (AHD) and the height of the SMH obtained from optical coherence tomography.

**Results:**

In total, 52 eyes from 52 patients were enrolled in this study; 16 eyes (30%) showed breakthrough VH. The proportions of patients with a current smoking history were 75.0% in the VH group and 22.2% in the non-VH group (p = 0.010). Other factors did not differ significantly between the two groups. The proportion of cases with accompanying sub-RPE hemorrhage was 50.0% and 58.3% in the VH and non-VH groups, respectively (p = 0.763). The AHD (p = 0.001) and SMH height (p < 0.001) were significantly greater in the VH group. In a receiver operating characteristic curve analysis, the cut-off values of AHD and SMH height were 20.1 and 1208 μm, respectively. According to logistic regression analysis, when the AHD and SMH height were greater than the individual cut-off values, the odds ratio of VH increased by 10.286 fold (95% confidence interval [CI], 2.452–43.148; p = 0.001) and 75.400 fold (95% CI, 7.991–711.441; p < 0.001), respectively, with respect to their respective reference groups (less than the cut-off value). Among the significant factors associated with VH occurrence, including current smoking, AHD, and SMH height, only current smoking and SMH height were found to be significant in multiple regression analysis (p = 0.040, 0.016).

**Conclusions:**

The incidence of breakthrough VH was significantly higher in those with current smoking status and for SMH with a larger AHD and greater height. The height of the SMH was more predictable of the possibility of VH than AHD.

## Introduction

Submacular hemorrhage (SMH) is a rare complication of age-related macular degeneration (AMD) and can cause a sudden loss in visual acuity and poor visual prognosis if it remains untreated [1–4]. Hemorrhage between retinal photoreceptors and the retinal pigment epithelium (RPE) can cause damage to the retina due to toxicity and the barrier effect. Iron and hemosiderin from red blood cells are toxic to the retina, whereas fibrin clots may damage the photoreceptor layer [5, 6]. In addition, blood clots act as barriers between the retina and choroid, thus inhibiting the transfer of oxygen and nutrients [3]. Subretinal fibrosis and disciform scarring from a SMH may cause serious visual impairment [5, 6].

The treatment of SMH generally consists of intravitreal anti-VEGF injections, intravitreal tissue plasminogen activator (tPA) injections, and intravitreal gas injections (pneumatic displacement), either alone or in combination. Vitrectomy with pneumatic displacement, with or without tPA, is generally reserved for more severe cases [3, 7–12].

Injection treatments are less invasive than vitrectomy, and there have been several reports of visual improvement after intravitreal anti-VEGF injections in AMD patients with SMH [13–15]. Intravitreal injections of anti-VEGF alone may decrease the incidence of recurrent bleeding but do not address the toxicity to the photoreceptors and RPE from heme and its hemolytic products. Other reports have indicated greater visual improvement with combined intravitreal anti-VEGF, tPA, and gas injections, relative to intravitreal anti-VEGF injections alone [16, 17]. Therefore, if the SMH is medium size, pneumatic displacement is usually recommended. However, complications such as vitreous hemorrhage (VH), SMH recurrence, retinal detachment, and endophthalmitis can occur after treatment, with VH as the most common complication (8–36%) [18–21].

Thus, we investigated factors that could predict VH occurrence after an intravitreal tPA and gas injection in AMD patients with SMH.

## Methods

This was a retrospective case study. The protocol was approved by the Institutional Review Board of Kosin University Hospital and adhered to the tenets of the Declaration of Helsinki. The requirement for obtaining informed patient consent was waived due to the retrospective nature of the study.

We reviewed the medical records of naïve patients who visited the Department of Ophthalmology of Kosin University Gospel Hospital from January 2010 to July 2018 and who received intravitreal tPA (Actilyase^®^, Boehringer Ingelheim GmbH, Ingelheim am Rhein, Germany) and octafluoropropane (C_3_F_8_; Metheson Tri-Gas, Montgomeryville, PA, USA) injections based on a diagnosis of AMD with SMH. Patients treated previously for AMD or SMH were excluded. Electronic medical records were searched using the keywords “Intravitreal tPA + octafluoropropane (C_3_F_8_) gas injection”, “Submacular hemorrhage”, and “Age-related macular degeneration”. Records were reviewed manually. Cases where the SMH was secondary to trauma and those of retinal arterial macroaneurysm, Terson syndrome, and unidentifiable causes were excluded.

The diagnosis was determined by slit-lamp examination and dilated fundus examinations, fundus photography (Kowa Nonmyd 7; Kowa Co. Ltd., Nagoya, Japan), spectral domain (SD)-optical coherence tomography (OCT; HRA Spectralis+OCT, Heidelberg Engineering, Heidelberg, Germany), and fluorescein angiography and indocyanine green angiography (FA/ICGA; HRA Spectralis, Heidelberg Engineering Inc., Heidelberg, Germany).

When the SMH was too severe and the condition was not conclusive but highly suspicious of AMD at the initial visit, treatment was performed first followed by OCT and FA/ICG examinations at subsequent visits to confirm choroidal neovascular lesions. Patients with histories of previous AMD treatment (intravitreal injections or photodynamic therapy) or retinal diseases with a risk of bleeding (e.g., diabetic retinopathy, retinal vascular occlusion, and previous vitrectomy) were excluded, as were patients whose OCT or FA/ICGA results were lost. Distorted OCT images (e.g., inverted images) due to an excessively thick SMH were also excluded.

The procedures were performed in an operating room. All pupils were dilated with phenylephrine hydrochloride and tropicamide ophthalmic solution (Mydrin-P^®^; Santen Pharmaceutical Co., Ltd., Osaka, Japan). After the instillation of 0.5% proparacaine hydrochloride ophthalmic solution (0.5% Alcaine^®^; Alcon, Fort Worth, TX, USA), the eyes were sterilized with 5% betadine. Periorbital skin was disinfected with 10% betadine, and disposable aseptic pouches were used in the procedure.

After exposing the cornea and conjunctiva with a speculum, the surfaces were irrigated with 5% betadine and sterile normal saline. Before injection, anterior chamber paracentesis was performed with a 30-gauge needle to lower the intraocular pressure. tPA (50 μg/0.05 mL) and pure octafluoropropane gas (0.3 mL) were injected into the vitreous at 5-min intervals.

Postoperatively, all patients were instructed to use 0.5% moxifloxacin hydrochloride ophthalmic solution (0.5% Vigamox^®^; Alcon), four times per day, and ofloxacin (3 mg/g) ophthalmic ointment (Tarivid Ophthalmic Ointment^®^; Santen Pharmaceutical Co., Ltd.) once per day. They were also instructed to maintain a prone position for 1–2 weeks based on the SMH status. After pneumatic displacement, anti-VEGF treatments were performed as three monthly loadings and subsequent as-needed injections.

**Fig 1:**
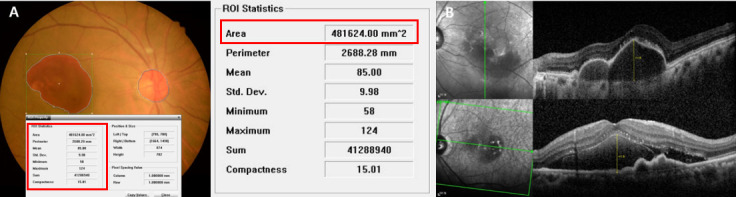
Submacular Hemorrhage (SMH) parameters. (A) Area ratio of the SMH to the optic disc (AHD) measured using the region of interest in the picture archiving and communication system. We used the area value. (B) SMH height measured with optical coherence tomography (OCT). We measured the height of the highest portion of the SMH.

We regarded VH within 2 weeks after the intravitreal tPA and octafluoropropane gas injection as a complication after treatment. The eyes were then classified into breakthrough VH and non-VH groups. We reviewed the age, sex, best-corrected visual acuity (BCVA), type of AMD, accompanying sub-RPE hemorrhage, history of cataract surgery, history of hypertension and diabetes mellitus, history of alcohol use and smoking, and history of antiplatelet or anticoagulant medication. The size of the SMH was analyzed in two ways. The area ratio of SMH to the optic disc (AHD) was calculated using statistical measurements of the region of interest in the picture archiving and communication system (PACS; Maroview^®^ ver. 5.4, Marotech, Seoul, South Korea). The area of the SMH divided by the area of the disc was defined as the AHD. Using OCT, the height of the SMH was determined using the PACS measurement function as the vertical height from the basal portion to the apex of the hemorrhage in the largest cross-sectional area of the SMH among the horizontal and vertical sections of the OCT image (Fig 1). Cases in which the images were distorted by an excessively thick SMH were excluded.

### Statistics

A statistical analysis was performed using SPSS statistical software, version 22.0 (IBM Corp., Armonk, NY, USA). The chi-square test was used for statistical analyses of the relationships between discontinuous variables and VH occurrence. The Mann-Whitney U test was used to compare mean values of continuous variables; p < 0.05 was considered to be statistically significant. Receiver operating characteristic curves (ROC curves) were applied to confirm the usefulness of variables as a predictor of VH occurrence. Logistic regression analysis was used to obtain the odds ratio (OR) of VH occurrence according to the AHD and SMH height.

## Results

### Predictive factors: Baseline characteristics

In total, 52 eyes from 52 patients were enrolled in this study; 16 eyes (30.8%) had a breakthrough VH. There was no statistically significant difference between the two groups with respect to age, sex, BCVA, history of hypertension and diabetes mellitus, history of alcohol use, history of antiplatelet or anticoagulant medication, duration of symptoms, accompanying sub-RPE hemorrhage, or AMD type. Table 1 shows that current smoking status differed significantly (p = 0.010) between the VH group (12 of 16 patients; 75.0%) and the non-VH group (8 of 36 patients; 22.2%). Vitrectomy was performed in nine patients in the VH group (56.3%). The mean interval between VH occurrence and surgery was 3.2 (± 1.6) weeks.

**Table 1:** Clinical characteristics of the breakthrough VH group and non-breakthrough VH group. Breakthrough VH is defined as VH occurring within 2 weeks after an intravitreal tPA and octafluoropropane gas injection.

	VH group (n = 16)	Non-VH group (n = 36)	p-value
Age (years)	63.1 (± 9.4)	66.7 (± 6.3)	0.290†
Sex (n, %)			0.940‡
Male	10 (62.5%)	22 (61.1%)	
Female	6 (37.5%)	14 (38.9%)	
BCVA at initial visit (logMAR)	1.31 (± 0.58)	0.97 (± 0.61)	0.190†
Intraocular pressure (mmHg)	13.3 (± 3.2)	13.0 (± 3.2)	0.730†
Hypertension (n, %)	8 (50.0%)	18 (50.0%)	0.080‡
Diabetic mellitus (n, %)	6 (37.5%)	12 (33.3%)	0.490‡
Anti-platelet or anti-coagulant agent (n, %)	2 (12.5%)	10 (27.8%)	0.301⁑
Alcohol (n, %)	4 (25.0%)	8 (22.2%)	0.270‡
Current smoking (n, %)	12 (75.0%)	8 (22.2%)	**0.010**‡
Duration of symptom (days)	8.7 (± 8.8)	8.4 (± 7.3)	0.901†
Accompanying sub-RPE hemorrhage			0.763‡
(+)	8 (50.0%)	21 (58.3%)	
(-)	8 (50.0%)	15 (41.7%)	
Subtype of AMD (n, %)			0.298⁑
CNV	2 (12.5%)	12 (33.3%)	
PCV	14 (87.5%)	24 (66.7%)	

^†^: Mann-Whitney U test;

^‡^: Chi-square test;

^⁑^: Fisher’s exact test.

BCVA, best-corrected visual acuity; RPE, retinal pigment epithelium; VH, vitreous hemorrhage; tPA, tissue plasminogen activator.

Boldface numbers indicate statistically significant differences at p < 0.05.

### Predictive factors: Image parameters

**Fig 2:**
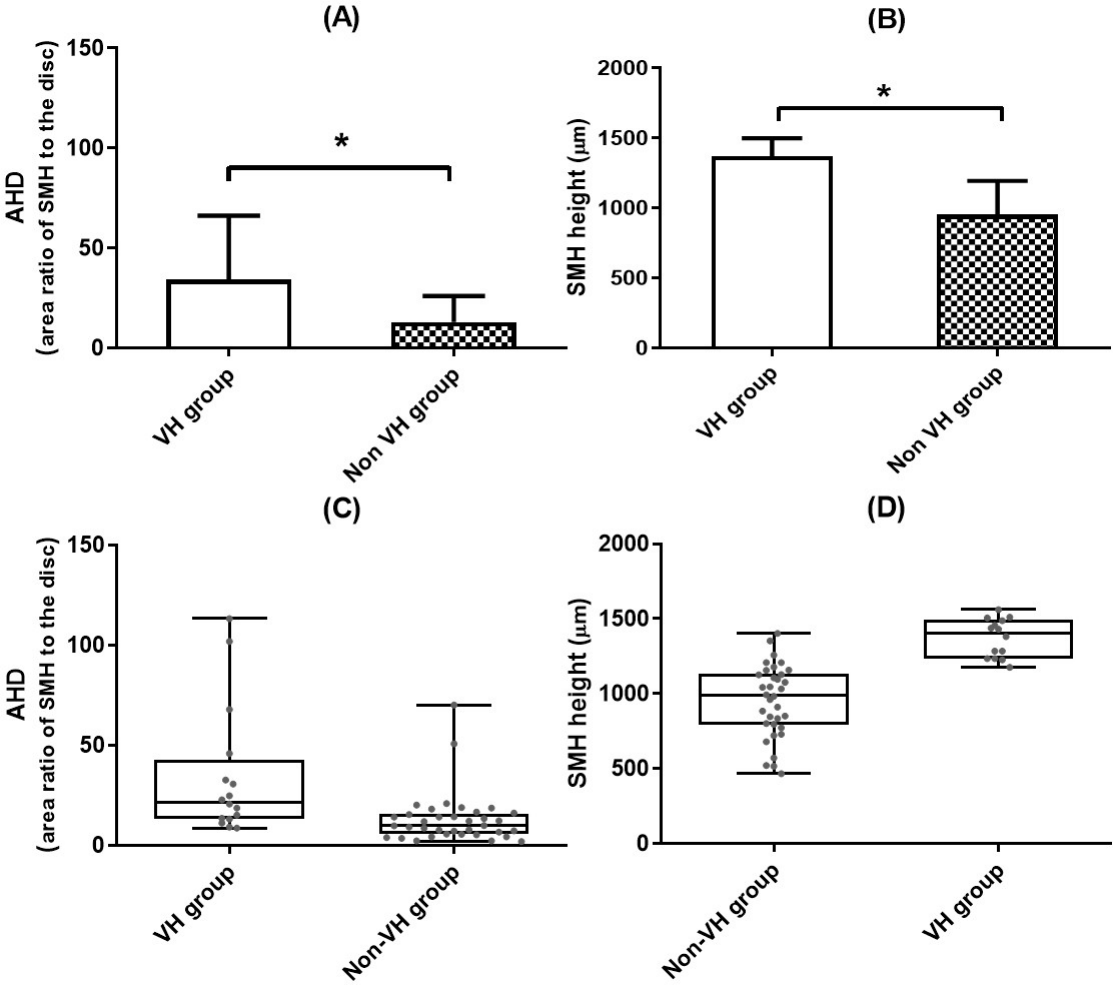
Comparison of the area ratio of the SMH to the optic disc (AHD) and SMH height between Vitreous Hemorrhage (VH) and non-VH groups. (A, B) Both AHD and SMH height were significantly greater in the breakthrough VH group. The error bar represents the standard deviation. (C, D) The distributions of the parameter values are shown in the box and scatter plots; the AHD values are more varied than those of SMH height. The boxes represent the 25% to 75% (lower to upper) quartiles; the lines in the boxes are the medians, and the whiskers indicate variability (minimum and maximum values). (*: p< 0.05, Mann-Whitney U test).

The average AHD and SMH height were 19.5 (± 22.9) and 1074.9 μm (± 287.3 μm), respectively. The mean AHD was 34.2 (± 32.4) in the VH group, which was significantly larger than that in the non-VH group (13.0 ± 13.0, p = 0.001). The height of the SMH was 1371.6 ± 128.0 μm in the VH group and 952.8 ± 241.6 μm in the non-VH group; the difference was statistically significant (p < 0.001). However, the distribution of AHD values was more varied compared to SMH height (Fig 2).

**Fig 3:**
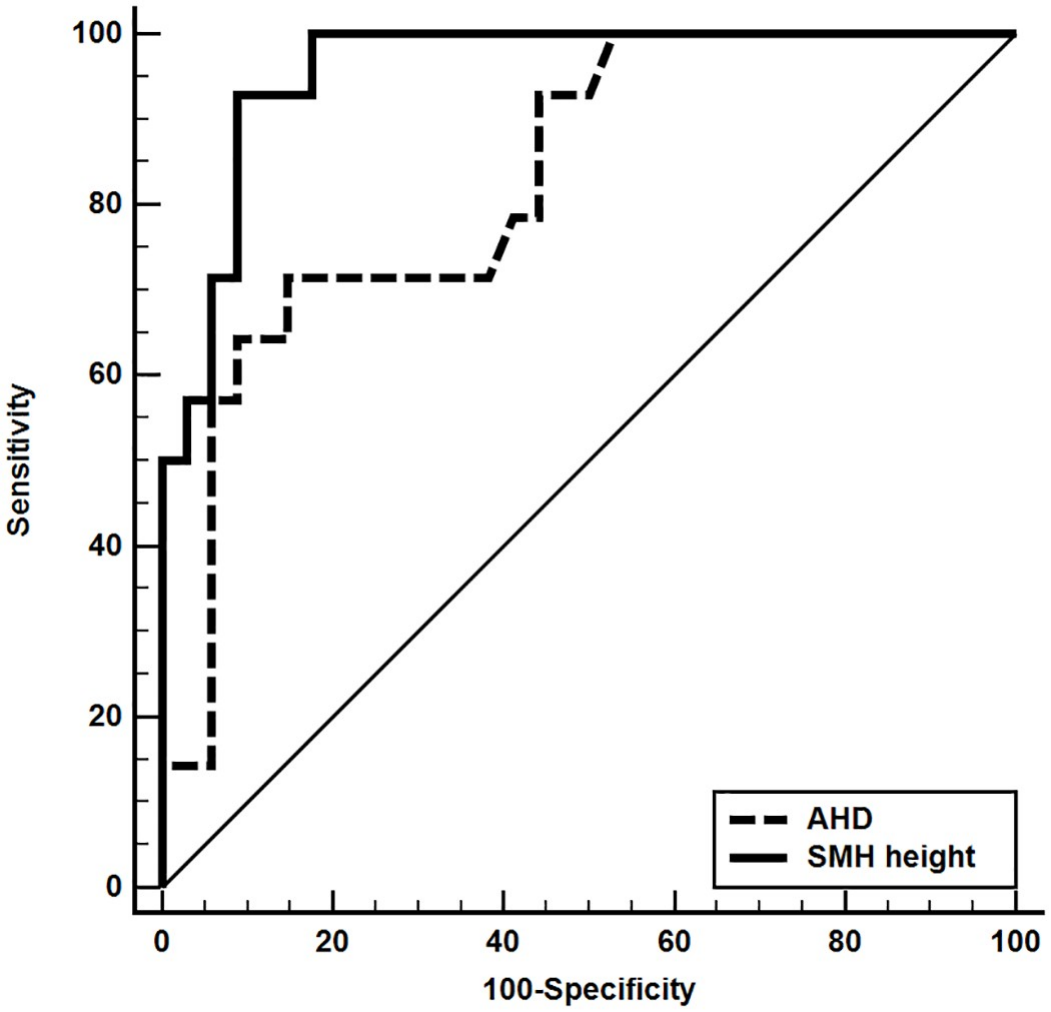
Receiver operating characteristic curves of AHD and SMH height; the areas under the receiver operating characteristic curve are 0.829 and 0.958, respectively.

The areas under the ROC curves (AUCs) of the AHD and SMH height were 0.829 (95% confidence interval [CI], 0.693–0.922) and 0.958 (95% CI, 0.856–0.994). The AMD and SMH height cut-off values were 20.1 (sensitivity, 56.2%; specificity, 91.7%) and 1208 μm (sensitivity, 92.9%; specificity, 91.2%), respectively (Fig 3).

### OR of VH recurrence according to AHD and SMH height

We used logistic regression analysis to obtain the OR of VH recurrence according to AHD and SMH height. We divided the AHD and SMH height into two groups according to the cut-off values from the ROC analysis of 20 and 1200 μm, respectively. In the group with AHD ≥ 20, the OR was 10.286 (95% CI, 2.452–43.148; p = 0.001), with respect to the reference group with an AHD < 20. In the case of SMH height, the OR of the group ≥ 1200 μm was 75.400 (95% CI, 7.991–711.441; p < 0.001) with respect to the reference group with SMH height < 1200 μm (Table 2).

**Table 2:** Logistic regression analyses for breakthrough vitreous hemorrhage prediction.

	Classification (proportion, %)	B	OR (95% confidence interval)	p-value
AHD	< 20.0 (75.0%)	Reference		
	≥ 20.0 (25.0%)	2.331	10.286 (2.452–43.148)	**0.001**
SMH height (μm)	<1200 (62.5%)	Reference		
	≥1200 (37.5%)	4.323	75.400 (7.991–711.441)	**< 0.001**

OR, odds ratio; AHD, area ratio of the SMH to the optic disc; SMH, submacular hemorrhage.

The reference values of the classification are the cut-off value from receiver operating characteristic curves analysis.

Boldface numbers indicate statistically significant differences at p < 0.05.

The significant factors, including current smoking, AHD, and SMH height, were analyzed by multiple regression analysis. Among these factors, multiple regression analysis showed current smoking (OR, 16.753; 95% CI, 1.141–245.981) and SMH height (OR, 1.017; 95% CI, 1.003–1.030) as significant (p = 0.040 and p = 0.016, respectively) (Table 3).

**Table 3:** Multiple regression analyses for three significant factors including current smoking and area ratio of Submacular Hemorrhage (SMH) to the optic disc, the height of the SMH.

	B	OR (95% confidence interval)	p-value
Current smoking^a^	2.819	16.753 (1.141–245.981)	**0.040**
AHD^b^	0.009	1.009 (0.946–1.076)	0.783
SMH height^c^	0.170	1.017 (1.003–1.030)	**0.016**

OR, odds ratio; AHD, area ratio of SMH to the optic disc; SMH, submacular hemorrhage.

^a^Categorical variable;

^b,c^ continuous variables.

Boldface numbers indicate statistically significant differences at p < 0.05.

### Complications associated with treatment

Complications included one case of rhegmatogenous retinal detachment in the VH group. There were no other complications, such as elevated IOP or endophthalmitis, after tPA or gas injection treatment.

## Discussion

SMH is a rare complication of AMD, which causes a sudden loss in visual acuity and poor visual prognosis if it remains untreated [2–4]. Bennet et al. [2] reported a mean visual acuity of 20/1700 three years after SMH was associated with AMD. Treatment of SMH is divided into non-surgical treatment (e.g., intravitreal injections) and surgical treatment (e.g., pars plana vitrectomy). With respect to non-surgical treatment, intravitreal gas injection alone, intravitreal gas with tPA injection, and intravitreal anti-VEGF injection have shown favorable results regarding improvement in visual acuity [4, 13–16, 18–20, 22–24]. These therapies, which are less invasive than vitrectomy, are widely used in clinical practice. However, complications with non-surgical treatment have been reported such as VH, retinal detachment, and endophthalmitis. After intravitreal tPA and gas injection, VH is the most common (8–36%) among reported complications [18–20].

We investigated factors affecting VH occurrence after an intravitreal tPA and octafluoropropane gas injection in AMD patients with SMH. There were no statistically significant differences between the two groups in age, sex, BCVA, history of hypertension and diabetes mellitus, history of alcohol use, history of antiplatelet or anticoagulant medication, duration of symptom, or type of AMD.

Shin et al. [25] reported that polypoidal choroidal vasculopathy (PCV) subtype was a risk factor for breakthrough VH after anti-VEGF injection for SMH patients. However, there was no significant difference in the proportion of PCV between the two groups in the current study (p = 0.269), although the percentage of PCV was higher in the VH group than in the non-VH group (87.5% vs. 66.7%, respectively).

The proportion of patients who were current smokers was significantly higher in the VH group (p = 0.010). In a previous report, retinal and choroidal thicknesses were thinner in smokers than in non-smokers [26]. Thus, breakthrough VH may be associated with a thinner retinal layer in smokers. However, additional investigations, with more patients, are needed to confirm this hypothesis.

We investigated several parameters that may predict the risk of VH after intravitreal tPA with gas injection. Notably, both AHD and SMH height were significantly greater in the VH group. The mean AHD was 34.2 (± 32.4) and 13.0 (±13.0) in the VH and non-VH groups, respectively (p = 0.001). The mean height of the SMH was 1371.6 ± 128.0 μm in the VH group and 952.8 ± 241.6 μm in the non-VH group (p < 0.001).

Our findings are consistent with those of Wu et al.^21^ indicating that VH is more likely to occur after intravitreal tPA and gas injection in patients with a large SMH as indicated by the optic disc area (DA). They reported that a larger hemorrhage size was strongly associated with a significantly greater risk of VH; those with respective hemorrhages of 11–20, 21–30, and 31–40 DA were 9.46, 33.57, and 80.40 times more likely to develop VH, respectively, as those with a hemorrhage of 0–10 DA. Meanwhile, to date, no studies have specified the height of the SMH measured by SD-OCT as a potential risk factor of VH occurrence.

To evaluate the predictive capacity of individual parameters, we examined the ROC curves. The AUC of the SMH height was larger than that of the AHD. Although the area of the SMH was previously reported as an important factor in the occurrence of VH, in the current study, only SMH height was still significant (OR, 1.017; 95% CI, 1.003–1.030; p = 0.016) by multiple regression analysis, and AHD was not significant due to multicollinearity. Also, AHD values were highly variable compared to SMH height. The sensitivity and specificity were 92.9% and 91.2%, respectively, for the cut-off value of the height of the SMH. These results suggest that the height of the SMH measured by SD-OCT is more helpful in predicting VH occurrence after intravitreal tPA and gas injection than the size (i.e., area) of the SMH.

It is more intuitive and easier to measure the height of the SMH by SD-OCT using the viewer software, as opposed to measuring and calculating the area of the SMH with respect to the optic DA. Despite the SMH being excessive and dense, it may not be feasible to measure the height of the SMH by OCT. In the present study, due to the good tissue penetrance of SD-OCT, it was possible to measure a very thick SMH height.

Considering the cut-off value of each parameter, when the AHD is ≥ 20.0 and the height of the SMH is ≥ 1200 μm, the operator should consider the possibility of VH occurrence after intravitreal tPA and gas injection. According to logistic regression analysis, when AHD and SMH height were greater than the individual cut-off values, the OR of VH increased by 10.286 fold (95% CI, 2.452–43.148; p = 0.001) and 75.400 fold (95% CI, 7.991–711.441; p < 0.001), respectively, with respect to the associated reference groups (AHD < 20 and SMH height < 1200 μm, respectively).

This study has several limitations. As a retrospective analysis, there were inherent limitations in patient grouping. In addition, the number of patients was small and insufficient to perform parametric statistical analyses. Thus, the validity of the statistical analysis may be somewhat less due to the small sample size. A prospective study with more patients is needed to confirm the results of the present study. Despite its limitations, we believe that this study is meaningful in that it presents indicators of SD-OCT-guided parameters (e.g., the height of the SMH) that can predict VH development after SMH treatment, which was not reported so far. Although we regarded VH newly developed within 2 weeks after intravitreal tPA and octafluoropropane gas injection as a complication of treatment, the possibility of the natural course of AMD, or *de novo* VH not originating from SMH was not fully excluded. However, we excluded patients with diabetic retinopathy or retinal vascular occlusion history. Also, in all cases, the amount of SMH after intravitreal tPA and gas injection decreased. Thus, we could consider the VH to be a breakthrough-type complication following treatment. Another limitation is the absence of visual acuity data in the groups. For analysis of the association between VH occurrence and visual prognosis, a long-term study examining serial visual acuity changes may be necessary.

In conclusion, VH after intravitreal tPA and gas injection in patients with AMD associated with SMH was more likely to occur when the amount of SMH was greater. Although AHD and height of the SMH were all significantly associated with VH occurrence, SMH height was a stronger indicator of VH.
